# Process outcomes of a multifaceted, interdisciplinary knowledge translation intervention in aged care: results from the vitamin D implementation (ViDAus) study

**DOI:** 10.1186/s12877-019-1187-y

**Published:** 2019-06-25

**Authors:** Pippy Walker, Annette Kifley, Susan Kurrle, Ian D. Cameron

**Affiliations:** 1 Menzies Centre for Health Policy, University of Sydney, Charles Perkins Centre D17, Camperdown, NSW 2006 Australia; 2 John Walsh Centre for Rehabilitation Research, University of Sydney, Kolling Institute of Medical Research, Royal North Shore Hospital, St Leonards, NSW 2065 Australia; 3 NHMRC Cognitive Decline Partnership Centre, Hornsby Ku-ring-gai Health Service, Hornsby, NSW 2077 Australia

**Keywords:** Barriers, Falls, Frail older adults, Preventive medicine, Residential facilities, Vitamin D

## Abstract

**Background:**

Vitamin D supplement use is recommended best practice in residential aged care facilities (RACFs) for the prevention of falls, however has experienced delays in uptake. Following successful international efforts at implementing this evidence into practice, the ViDAus study sought to replicate this success for the Australian context. The aim of this paper is to report on the process outcomes of implementing this intervention.

**Methods:**

Forty-one RACFs were engaged in a multifaceted, interdisciplinary knowledge translation intervention. This focused on raising awareness to improve knowledge on vitamin D, and supporting facilities to identify barriers and implement locally devised strategies to improve the uptake of evidence based practice (EBP).

**Results:**

Staff members of participating facilities (*n* = 509 including nursing, care and allied health staff) were well engaged and accepting of the intervention, though engagement of servicing general practitioners (GPs) (*n* = 497) and pharmacists (*n* = 9) was poor. Facilities each identified between three and eight strategies focused on raising awareness, identifying residents to target for vitamin D and creating referral pathways depending upon their own locally identified barriers and capacity. There was variable success at implementing these over the 12-month intervention period. Whilst this study successfully raised awareness among staff, residents and their family members, barriers were identified that hindered engagement of GPs.

**Conclusions:**

The intervention was overall feasible to implement and perceived as appropriate by GPs, pharmacists, facility staff, residents and family members. More facilitation, higher-level organisational support and strategies to improve RACF access to GPs however were identified as important improvements for the implementation of vitamin D supplement use.

**Trial registration:**

Retrospectively registered (ANZCTR ID: ACTRN12616000782437) on 15 June 2016.

## Background

Research on the implementation of evidence-based practice (EBP) in the residential aged care setting is still an emerging field. Existing gaps in the literature have been recognised as presenting challenges for those focused on the uptake of EBP in residential aged care [1–3]. Unfortunately, there are no standardised approaches to identify what prevents the uptake of EBP, resulting in a reliance on ones best judgment [4]. As concluded by Grol [5], most approaches to implementing EBP are based on beliefs rather than evidence, highlighting the importance of establishing a good evidence base for effective knowledge translation.

Vitamin D supplement use has been identified internationally as a clinical practice that has experienced delays in uptake by residential aged care facilities (RACFs) [6]. There is robust evidence demonstrating the effectiveness and cost-effectiveness of vitamin D supplements in reducing falls and fall related harm in older people, as is reflected in both Australian and international best practice guidelines [7–11]. Despite this, there is a paucity of evidence for strategies that increase prescribing of vitamin D supplements for residents in Australian RACFs. Efforts to date have either worked with just one facility or organisation [12–14], or primarily focused on reducing falls rather than evaluating the implementation of vitamin D as a clinical practice [15, 16]. Although large international studies have demonstrated ability to implement vitamin D supplement use, the feasibility of replicating this for the Australian context is yet to be confirmed [17–21].

Despite a lack of evidence to inform the implementation of EBP, the promoting action on research implementation in health services (PARIHS) framework has been validated for use in the RACF setting as a framework of factors contributing to the success of implementing evidence-based nutrition and hydration practices [22–24]. This framework describes the interplay of evidence, context, and facilitation for the successful implementation of EBP [25]. The ViDAus study applied these principles and drew on the current evidence for effect [2] to select a suite of suitable strategies given the Australian aged care context and availability of resources.

Sufficient ‘evidence’ for implementation can be drawn from research, clinical experience, patient experience and local data or information, so the ViDAus study provided education on the available research for the use of vitamin D supplements and conducted medication chart audits to feedback local data on the use of vitamin D. ‘Context’ as characterised by culture, leadership, and evaluation was reflected in this trial through the recruitment of a local champion at each facility and engaging in local evaluation of barriers to and uptake of vitamin D supplements. Lastly, ‘facilitation’ as defined in the PARIHS framework by purpose, role, skills and attributes has been reflected in this intervention through external facilitation of quality improvement planning by a project officer [25].

The primary aim of the ViDAus study was to increase the proportion of residents in each participating facility prescribed an adequate dose (≥800 IU/day) of a vitamin D supplement. Other related medication chart variables such as calcium, osteoporosis medication use and the total number of prescribed oral medications, in addition to falls rates by participating facility were also evaluated. The effect on these outcome measures will be reported elsewhere.

The aim of this paper is to report on process outcomes of the ViDAus study evaluating the feasibility of this multifaceted, interdisciplinary knowledge translation intervention for the implementation of vitamin D supplement use in Australian RACFs. The purpose of reporting on these findings is to provide insights relating to how vitamin D supplementation was received by key stakeholders, important learnings regarding the feasibility of certain strategies for implementation in the RACF context, and the broader implications for consideration by policy makers and public health and health service organisations concerned with the health of older adults in RACFs.

## Methods

### Study design, participants and setting

The study followed a pragmatic non-randomised stepped wedge cluster design as outlined in Table 1. Aged care organisations were recruited as a convenience sample through existing relationships with the research team who approached them to determine interest in participating. Central managers at each organisation responsible for the coordination of research nominated individual aged care facilities that had capacity to participate after considering their participation in other research for recruitment into the study. Clusters were individual facilities that did not provide care exclusively to bed bound or palliated residents, and had a baseline prevalence of adequate (≥800 IU) vitamin D supplement use below 80%. Facilities were allocated to one of two groups (wedges) with other facilities within their operational region to prevent contamination [15, 17]. Although facilities were recruited into the study to receive the intervention, the target group for implementation was all residents not currently prescribed an adequate dose (≥800 IU) of a vitamin D supplement.

**Table 1:** Stepped wedge trial design and implementation timeline

Month	Wedge 1 Facilities	Wedge 2 Facilities
0	*Medication Chart Audit 1*	
1–6	Intervention • Election of key contact person • Dissemination of introductory newsletter to stakeholders • Face-to-face education for facility staff • Provided printed educational resources • Dissemination of second newsletter to feedback baseline medication chart audit results to stakeholders	Control
6	*Medication Chart Audit 2*	
7–12	Intervention • Meeting and development of quality improvement plan • Dissemination of third newsletter to feedback six month medication chart audit results to stakeholders	Intervention (as per months 1–6 for wedge 1)
12	*Medication Chart Audit 3*	
13–18	Sustainment	Intervention (as per months 7–12 for wedge 1)
18	*Medication Chart Audit 4*	

The Australian residential aged care sector in 2014 provided 192,834 places across 2688 services, equating to an average of 72 beds per facility [26]. Facility staff consist of direct care staff (registered nurses, enrolled nurses, care workers) and non-direct care staff (managers, hospitality etc.), with nurses being university trained, and care workers holding a relevant certificate in aged care [27]. In 2012 there were 0.7 direct staff members in not-for-profit aged care facilities per operational place [27].

General practitioners (GPs) and pharmacists are visiting health care providers who are not usually employed by RACFs. For this reason, the frequency of their visits is at their own discretion with many servicing several sites or working concurrently in general practice. A 2017 survey of GPs indicated that they average 8.6 visits per month (range 0–65 visits) and see an average of 6.6 patients per visit (range 0–55 patients) [28]. Residential medication management reviews (RMMRs) are available annually to residents and are conducted by pharmacists in collaboration with GPs for residents that would benefit from review. A 2009 survey of pharmacists reported an average of 5.4 visits to conduct RMMRs in the last 12 months (3.9 for facilities with up to 40 beds up to 6.5 for facilities with over 80 beds) with most (57%) conducting between 6 and 20 reviews in a single visit [29].

### Intervention

The intervention was delivered over a 12 month period, with each group starting six months apart as shown in Table 1. The trial ran from December 2015 to June 2017. A project officer was employed to coordinate the implementation of strategies concerning key stakeholders, including facility staff members, servicing GPs and pharmacists and the residing aged care residents and their relatives. A description of the included strategies is provided in Table 2, with detail concerning the timeline of implementation provided in Table 1. For this intervention the main target group were the facility staff, which refers to RACF employees (managers, nurses, care workers etc.). As the frequency of site visits from GPs and pharmacists is variable, it was only feasible to provide access to online materials, written correspondence and encourage follow up from facility staff rather than face-to-face education.

**Table 2:** Implementation strategies

Appointment of a local champion	Each participating site nominated a key contact person who would liaise with the study project officer to coordinate project related activities, drive implementation onsite and provide feedback to the project officer.
Educational outreach	The study project officer delivered a face-to-face education session to staff at each participating aged care facility over one to two visits within the first three months of the project. In some instances, residents were also invited. The learning objectives of this session was to understand; the function of vitamin D, the causes and extent of vitamin D deficiency in Australia, the effects of vitamin D deficiency and groups that are at risk and to identify sources and understand recommended intakes of vitamin D and calcium. The educational session was developed by the research team using the most up to date evidence and was also made available online: http://sydney.edu.au/medicine/cdpc/resources/facility-staff.php.
Educational resources	Educational posters, brochures and study pens were provided to each site to circulate to their staff, residents and family members. These resources and a short educational video were available online: sydney.edu.au/medicine/cdpc/resources.
Audit and feedback	Medication charts audits were provided by servicing pharmacists at six month intervals to establish the proportion of residents at each participating facility that were prescribed an adequate dose of vitamin D (Table 1). De-identified reports were provided to the investigators who analysed the data to establish the prevalence of vitamin D supplement use at the end of each six month period (4 audits in total). This information was reported back to key stakeholders via published study newsletters throughout the project period.
Expert opinion leader	All communication with GPs and pharmacists (via fax or email) was signed by an expert geriatrician to add credibility to the information being provided as this strategy has some observational evidence of effect [30] and direct contact was not feasible. This included communication regarding audit results and the availability of online resources to encourage the consideration of vitamin D for their residents.
Facilitated quality improvement	In the second six months of the intervention the project officer met with the leadership team at each site to discuss barriers to implementation and feasible strategies to improve the uptake of vitamin D supplement use. This information was summarised into a quality improvement plan for each site. Since no more than two separate visits to each site was feasible for this study, progress on the implementation of derived strategies was discussed with the nominated champion during one to two follow up phone calls from the project officer.

### Outcomes

Process outcomes relate to participant retention, the delivery of the intervention with respect to the intended timeline, stakeholder engagement, the identification of local barriers and implementation of devised strategies as part of quality improvement (QI), and the perceived appropriateness of the intervention. Outcomes relating to the overall effect of the intervention on vitamin D supplement use prevalence will be reported elsewhere.

Stakeholder engagement measures included the nomination of a key contact by participating facilities, attendance at face-to-face education sessions, attendance at the QI planning meeting, and survey response rates. Changes in knowledge, confidence, professional practice and access of the information provided to stakeholders were evaluated through surveys. Surveys were administered to all stakeholder groups (GPs, pharmacists and facility staff) at baseline and following the 12 month intervention. In addition to this, a similar survey was provided to facility staff directly before and after face-to-face education sessions.

These surveys were developed specifically for this trial by the investigators. Questions were derived from the literature on the likely barriers to implementation [31], and to evaluate the key learnings of content delivered in education sessions. Prior to implementation the surveys were pilot-tested and validated by eight content experts, including representatives from the participating aged care organisations (*n* = 2), registered nurses (*n* = 2), dietitians (*n* = 2) and researchers with a background in pharmacy (*n* = 2) who were asked to provide feedback on the content, readability and relevance of the questions. Surveys were administered to participating facility staff via the key contact or project officer, and to GPs and pharmacists via fax or email.

The surveys also sought to evaluate the perceived appropriateness of both the intervention overall and the delivery of face-to-face education. Residents and family members had the opportunity to provide feedback on the educational resources that were made available to them via a short separate survey. All survey responses were voluntary and anonymous. Key contacts provided feedback on the appropriateness of the intervention, updates on the implementation of devised QI strategies, and perceived barriers to implementation during a follow up phone call at the end of the intervention period.

## Results

### Participants and timing of intervention delivery

Forty-one facilities (17 in wedge 1 and 24 in wedge 2) across four not-for-profit aged care organisations in NSW (*n* = 31 facilities across 2 organisations) and SA (*n* = 10 facilities across 2 organisations) completed the intervention, which together support approximately 3400 residents (approx. 1500 in wedge 1 and 1900 in wedge 2) with an average bed capacity per facility of 83 beds (range 30–289). Average baseline prevalence of adequate (≥800 IU/day) vitamin D supplement use was 56.2% (95%CI 51.4–61.0) by facility (range 15.4–77.5%). Servicing GPs per site varied from 1 for every 2 residential beds, to 1 per 40 residential beds, with an average of 1 GP to service 5 residential aged care places (*n* = 497 across 41 facilities). There was one pharmacist per site that conducted medication reviews, with some pharmacists covering multiple sites (*n* = 9 pharmacists for 41 facilities). All 42 facilities that were recommended for recruitment met the inclusion criteria. One facility withdrew shortly following recruitment prior to commencing the intervention, due to their limited capacity to participate in research without a facility manager at that time.

The intervention was delivered as per Table 1, with a few exceptions. The first related to the collection and feedback of medication chart audit results. Due to delays in the turnaround time between the request for and provision of point prevalence data, feedback from the baseline and six month audit was not provided until the six month and one year point respectively. This was a result of the burden this task placed on pharmacists to provide this data as their software did not allow for de-identified reports to be produced. Secondly, there were some delays with conducting the QI planning meeting. These meetings were for most sites conducted at six months, with seven sites from the second wedge having this delayed until month eight or nine due to the availability of research or participating staff. Research staff availability was hindered by a reduction in the project officer’s contract from full time to two days per week prior to the commencement of sites in the second wedge. All of the face-to-face education sessions were provided in the first three months.

### Stakeholder engagement

Table 3 summarises the measures of engagement with key stakeholder groups involved in the study. All participating facilities nominated a key contact or ‘champion’ to liaise directly with the project officer. Sites nominated their site manager, deputy manager, director or deputy director of nursing as the key contact. Due to changes in capacity to undertake this role or staffing changes, 16 sites had at least one change to their key contact person during the intervention, with four sites having two changes, and one site having three changes. Just less than two thirds (58.5%) of key contacts attended both the face-to-face education session and the quality improvement planning meeting.

**Table 3:** Stakeholder engagement measures

Stakeholder Group	Measure	Value
Key Contacts	Number of sites that had a least 1 key contact change (%)	16/41 (39.0)
	Number that attended education (%)	27/41 (65.9)
	Number that attended QI planning meeting (%)	37/41 (90.2)
Facility Staff Members	Average number of staff attending education (range by facility)	12.4 (2–29)
	Average number of staff at QI planning meeting (range by facility)	2.8 (1–9)
Servicing GPs and pharmacists	Average number of survey responses for baseline and 12 month surveys combined (range by facility)	2.3 (0–8)

All participating facilities in some way provided an avenue for staff to receive education, with one organisation funding their staff to attend sessions outside of work hours to achieve greater attendance and minimise distractions to learning. Only two facilities could not allocate sufficient time for staff to come together as a group for education, with one arranging for ad hoc discussions in the lunch room throughout the day and another combining the project officer visit with staff handover.

A total of 509 staff members of participating facilities received face-to-face education on vitamin D, with the average and range by facility presented in Table 3. The proportion of staff attending education by facility bed capacity ranged from 2 to 40%, with an average of 16%. In addition to these sessions, in many cases residents were invited to sit in on the education sessions, of which a total of 51 accepted. A total of 113 staff members participated in QI planning, which typically included the key contact and other key staff members such as registered nurses, physiotherapists and QI staff.

The response rates of pre, and post face-to-face education surveys from participating staff members were reasonable, with 348/509 (68.4%) completing the pre-education questionnaire, and an additional 86 staff members that did not have time to complete the pre-education questionnaire completing the post education questionnaire (434/509; 85.3%). Respondents were similar for both surveys respectively being primarily care staff (51 and 53%), registered nurses (25 and 26%) and ‘other’ (24 and 21%), which included admin, hospitality and allied health staff. All measures of knowledge and confidence relating to vitamin D supplement use for falls prevention improved significantly following the education session, which was between 20 min to one hour in duration depending on the time available (Table 4). The same presentation was provided to all staff (see Table 2), though the longer sessions allowed for more question and discussion time.

**Table 4:** Changes to knowledge and confidence following facility staff face-to-face education

Question	Pre-Education (*n* = 348)	Post-education (*n* = 434)	*P* value
How much do you know about risk factors for low vitamin D levels? (proportion answering ‘considerable knowledge’ or ‘very knowledgeable’)	17.5	73.0	< 0.0001*
How confident are you at identifying residents at risk of vitamin D deficiency? (proportion answering ‘considerable confidence’ or ‘very confident’)	13.5	60.1	< 0.0001*
How much do you know about vitamin D supplements (benefits, risks, indicators, doses)? (proportion answering ‘considerable knowledge’ or ‘very knowledgeable’)	12.6	67.7	< 0.0001*
How confident are you about answering questions from residents or families about vitamin D supplements? (proportion answering ‘considerable confidence’ or ‘very confident’)	12.4	58.5	< 0.0001*

*Represents a significant difference of *p* ≤ 0.05, using the χ^2^ test

There was a very low response rate from GPs and pharmacists for both the baseline and 12 month survey, with 62/506 (12.1%) and 33/506 (6.5%) responding respectively. Pharmacists represented only a very small handful of the GP and pharmacist cohort invited to participate in study related surveys (~ 2%). Facility staff also had a low number of responses to this survey (177 and 28 responses for each survey respectively). Facility staff knowledge and confidence improved over the 12 month intervention and to a lesser extent for GPs and pharmacists (Table 5).

**Table 5:** Changes to knowledge and confidence following the 12 month intervention

Question	Facility Staff			GPs & Pharmacists		
	Baseline (*n* = 177)	After 12 months (*n* = 28)	*P* value	Baseline (*n* = 61)	After 12 months (*n* = 33)	*P* value
How much do you know about risk factors for low vitamin D levels? (proportion answering ‘considerable knowledge’ or ‘very knowledgeable’)	29.9	60.7	0.002*	82.3	91.0	0.4
How confident are you at identifying residents at risk of vitamin D deficiency? (proportion answering ‘considerable confidence’ or ‘very confident’)	26.6	53.6	0.007*	80.6	91.0	0.3
How much do you know about vitamin D supplements (benefits, risks, indicators, doses)? (proportion answering ‘considerable knowledge’ or ‘very knowledgeable’)	23.7	53.6	0.002*	77.4	78.8	~ 1
How confident are you about answering questions from residents or families about vitamin D supplements? (proportion answering ‘considerable confidence’ or ‘very confident’)	24.3	42.9	0.067	77.4	81.8	0.8

*Represents a significant difference of p ≤ 0.05, using the χ^2^ test

Facility staff indicated accessing the information provided as part of the study (e.g. brochures, posters, online webinar and other written resources), with only 4.4% (1/23) indicating they did not access any study related material. Conversely, 48.5% (16/33) of GPs and pharmacists indicated not accessing any of the information made available. Survey respondents that indicated they discussed or considered vitamin D supplements for residents in their professional practice either ‘most of the time’ or ‘always’ increased for facility staff from 23.4 to 55.6% after 12 months, however did not change for GPs and pharmacists (70.5% at baseline and 69.7% after 12 months).

### Locally devised implementation strategies

All participating facilities discussed locally appropriate strategies with the project officer. Each facility identified between three and eight strategies specific to the needs of their site. Strategies have been grouped into three broad categories. These focused on either (1) increasing or maintaining knowledge or awareness of vitamin D among facility staff members, residents and family members, (2) identifying residents that were suitable for the recommendation of vitamin D, and (3) implementing referral pathways or targeting prescribers for the consideration of vitamin D supplementation (Table 6). At the individual facility level an average of 3.7 strategies were implemented per site (range 0–6), which equates to an average of 65.5% of the strategies initially devised in the QI plan (range 0–100%). Two facilities did not implement any of the strategies they originally set out to, due to competing priorities and inconsistent leadership (4 key contact person changes across the two sites in 12 months). Regarding GP engagement, 33 facilities (81%) identified the need to engage with GPs in at least one way, either to raise general awareness (22%) or to follow up regarding individually identified residents (44%), with six (15%) engaging GPs in both ways. The nominated key contact person at each site led the implementation of identified quality improvement strategies.

**Table 6:** Quality improvement strategies identified and implemented by participating sites

Strategy	Facilities that identified (%)	Facilities that implemented (%)
Knowledge/awareness of vitamin D		
Face-to-face education for residents	28/41 (68)	21/28 (75)
Information and resources emailed to families	14/41 (34)	10/14 (71)
Making resources available onsite (in addition to study posters and brochures e.g. newsletters)	12/41 (29)	12/12 (100)
Face-to-face education for families	12/41 (29)	9/12 (75)
Adding vitamin D to staff meeting agendas	11/41 (27)	9/11 (82)
Embedding ongoing education for staff into the workplace *(*e.g. *adding links or information to online portals)*	11/41 (27)	5/11 (46)
Additional face-to-face education for staff	3/41 (7)	2/3 (67)
Identification of residents suitable for vitamin D		
Conducting a one-off audit to identify residents not currently prescribed, and potentially suitable for vitamin D	29/41 (71)	21/29 (72)
Adding vitamin D to online or hard copy assessment forms *(*e.g. *falls risk ax, admission or case conference forms)*	23/41 (56)	0/23 (0)
Implementing an unwritten process or procedure to identify residents suitable for vitamin D *(*e.g. *staff to remember to check on admission or during a case conference/ care plan review)*	20/41 (49)	16/20 (80)
Arranging an ongoing audit to identify residents suitable for follow up (either internally or as a request to pharmacy)	1/41 (2)	0/1 (0)
Referral pathways		
Follow up with GPs regarding specific residents that have been identified as potentially suitable for vitamin D	23/41 (56)	14/23 (61)
General follow up with pharmacists to raise awareness	17/41 (41)	11/17 (65)
General follow up with GPs to raise awareness	16/41 (39)	16/16 (100)
Follow up with physiotherapists for support	3/41 (7)	2/3 (67)
General follow up with nurse practitioners to raise awareness	1/41 (2)	0/1 (0)

### Barriers to implementation

Key contacts at each participating facility identified between two and nine potential barriers to implementation at the end of the intervention period (Table 7). There was less of a focus placed on enablers to implementation with some staff mentioning points such as improved knowledge, having good relationships with and support from GPs and pharmacists, support from family members, peer pressure from other residents to consider vitamin D and ongoing internal audits to identify residents that are not prescribed an adequate dose of vitamin D for follow up.

**Table 7:** Summary of identified barriers of participating key contacts

Summary of barriers in order of prevalence	Number of facilities (%)
Resident & family beliefs or attitudes *(*e.g. *related to polypharmacy, cost or cultural barriers)*	34 (83)
GP beliefs & attitudes *(*e.g. *related to clinical evidence, polypharmacy, blood tests or sunlight exposure)*	26 (63)
Suitability of residents for vitamin D *(*e.g. *due to medical contraindication, vitamin D sufficiency, immobility, perceived need for vitamin D relating to cognition, sunlight exposure or changing care needs)*	20 (49)
Competing priorities/ time/ capacity to implement *(*e.g. *due to staff shortages, other projects focused on polypharmacy or medication simplification, lack of funding to support prevention or competing priorities such as accreditation & renovation)*	20 (49)
Resident and family knowledge or understanding	17 (42)
Awareness/ process/ prompt for staff	17 (42)
Resident behaviours *(*e.g. *medication refusal due to dementia, swallowing difficulties or mental health)*	13 (32)
Contact with general practitioners *(*e.g. *due to having many GPs, or GPs that do not regularly visit)*	12 (29)
Staff turnover	12 (29)
Resident turnover	10 (24)
Leadership/ culture/ motivation to change *(from both the local management and organisation levels)*	8 (20)
GP knowledge *(*e.g. *related to current guidelines and available vitamin D formulations)*	7 (17)
Pharmacist attitudes & beliefs	6 (15)
Staff knowledge	2 (5)
No obvious way to demonstrate the benefits of vitamin D	1 (2)

Stakeholder beliefs and attitudes were commonly reported as a hindrance to successful implementation or uptake of vitamin D supplement use (Table 7). Changes in attitudes and beliefs surrounding vitamin D supplement use were evaluated in both the baseline and 12 month survey (Table 8). Changes to attitudes and beliefs among facility staff were more apparent following face-to-face education, with disagreement with the statement that vitamin D is not important for falls prevention changing from 56.9 to 79.0% (*p* < 0.0001), agreement that vitamin D should be recommended for all residents unless medically contraindicated changing from 66.4 to 89.6% (*p* < 0.0001), disagreement that vitamin D supplement use will contribute to polypharmacy changing from 48.6 to 67.5% (*p* < 0.0001), and agreement that vitamin D is affordable for most residents changing from 62.6 to 69.8% (*p* = 0.04) of facility staff following education compared with the survey completed immediately prior to the session.

**Table 8:** Changes to attitudes and beliefs

Statement	Facility Staff			GPs & Pharmacists		
	Baseline (*n* = 177)	After 12 months (*n* = 27)	P* value	Baseline (*n* = 61)	After 12 months (*n* = 33)	P* value
**Vitamin D supplements are not a particularly important falls prevention strategy** (proportion answering ‘disagree’ or ‘strongly disagree’)	65.0	74.1	0.4	86.9	87.9	~ 1
**Vitamin D supplements should be recommended for people living in residential aged care facilities unless there is a medical reason to withhold vitamin D** (proportion answering ‘agree’ or ‘strongly agree’)	71.2	81.5	0.3	83.6	84.8	~ 1
**Vitamin D supplement use will unnecessarily increase the total number of medications** (proportion answering ‘disagree’ or ‘strongly disagree’)	52.0	70.4	0.1	70.5	75.8	0.7
**Vitamin D supplements are affordable for the majority of residents** (proportion answering ‘agree’ or ‘strongly agree’)	54.8	63.0	0.5	62.3	81.8	0.08

*Calculated using the χ^2^ test, all values not statistically significant

### Perceived appropriateness

Feedback specific to the face-to-face education from participating facility staff was largely positive, with over 95% agreeing the information provided was easy to understand, appropriate for their level of knowledge, important that vitamin D education is provided to aged care facility staff members and that the education provided would be useful for future staff members.

A total of 52 surveys were received from residents or family members, of which 49 (94.2%) reported that the information was easy to understand, 43 (82.7%) reported that the information helped them to understand more about the importance of vitamin D, and 48 (92.3%) reported that they felt this information is important for other residents, their friends and family members.

Feedback from stakeholders received from the survey provided after the 12 month intervention indicated that most facility staff found the information provided as part of the study useful (78.3%), indicated that their professional practice had been influenced by the information received as part of the study (69.6%), and were confident that vitamin D supplements will be flagged as important for future residents at their facility (91.3%). The majority of pharmacists and GPs were also confident that vitamin D would be flagged for residents in the future (63.6%), however the majority neither agreed or disagreed as to whether they found the information useful (63.6%) or felt their professional practice was influenced by the information provided (57.6%).

## Discussion

This study demonstrated both strengths and areas for improvement in the implementation of strategies focused on increasing the uptake of vitamin D supplements. Engagement of participating facility staff was generally satisfactory, with all sites nominating a key contact person who in most cases (58.5%) were present at both the onsite education session and the QI planning meeting (Table 3). The importance of this engagement was recognised by participating sites, with 20% of key contacts reporting leadership as a barrier to implementation (Table 7). This was reported by facilities that experienced changes to their key contact and were aware that the previous champion was not well engaged or participating in study related activities, indicating that the value of internal facilitation was realised [25].

Attendance at education sessions by on-site facility staff members was also reasonable. Although variable between sites (2–29), the mean number of staff members equated to 12.4 per site, which is similar to the 13.6 average engagement of staff per site in study meetings where education was provided as part of a similar implementation trial in Canada [21]. This could however be higher, with an average of 0.16 staff members per operational place receiving education, when figures show that in 2012 there were 0.7 direct staff members in not-for-profit aged care facilities per operational place [27]. The present cohort of staff members had a higher proportion of registered nurses than the Australian residential aged care workforce (26% vs. 15%), and a lower proportion of care workers (52% vs. 68%), which may influence the generalisability of educational outcomes [26].

Although survey responses from facility staff were low for the baseline and 12 month surveys (Tables 5 and 8), the priority for this group was the almost identical survey provided at the face-to-face education session to assess changes in knowledge and beliefs related to vitamin D supplementation. These results provide evidence of improvements in both knowledge and confidence related to the information presented, and acceptability of the sessions as both appropriate, and useful (Table 4). As a result of this, it was noted that very few (5%) key contacts felt that staff knowledge was a barrier to implementation at the end of the intervention (Table 7).

Just over half of participating sites (*n* = 21) provided education for their residents, and one quarter (*n* = 10) emailed study resources to family members (Table 6). Oudshoorn et al. [32] has reported previously that resident knowledge on vitamin D is associated with vitamin D sufficiency and indicated that the role of education should be investigated further. This study has been able to demonstrate that providing education to residents and family members, and making written resources available onsite are feasible strategies to implement, with between 71 and 100% of sites that included these strategies in their quality improvement plan being able to implement them during the intervention period, in addition to the positive feedback received from consumer survey responses (Table 6).

Despite the above-mentioned implementation strengths this study failed to achieve a clinically significant improvement in the prevalence of adequate (≥800 IU/day) vitamin D supplement use (overall average facility level increase of 3.9% over 12 months). Although these results are not the focus here, pertinent to the reported process outcomes, barriers were identified that may have hindered the transfer of this knowledge into the Australian RACF context.

The first of these was the limited facilitation provided by the study project officer due to an unanticipated reduced capacity. As key to implementation in RACFs, allowing more time for an external facilitator to provide support may have improved implementation, given some sites did not implement any of their planned strategies [23]. Similarly, the delays experienced in providing medication chart audit results to stakeholders may have had a negative effect on implementation, since physicians tend to be more accepting of timely feedback [33]. Achieving this was hindered by the time consuming nature the task placed on servicing pharmacists, who were completing this alongside their usual duties. Others have experienced similar difficulties, with ongoing follow up and reminders required to obtain the necessary information [21]. A new method to audit vitamin D supplement use that meets ethical requirements of ensuring all data remains de-identified may be the best way to overcome this barrier. Whilst being a cost-effective intervention at a population level and having previously been deemed inexpensive, cost was frequently reported as a perceived barrier to implementation (Table 7) [11, 14, 34, 35]. Previous implementation trials have acknowledged this, including in New Zealand where vitamin D was subsidised for residents nationally [19, 20, 36]. Further investigation is required to understand whether addressing the cost of vitamin D would result in improved uptake by residents in Australia.

Another barrier identified by key contacts was the perceived unsuitability of vitamin D. This was based on concerns of possible medical contraindications, vitamin D sufficiency, low falls risk and stage of life that a number of prescribers report as reasons for not prescribing vitamin D (Table 7). These concerns all relate to the changing care needs of residents living in RACFs, including increasing levels of dependency, increasing age upon admission and reduced length of stay, which may have implications for current best practice guidelines recommending vitamin D for all residents in RACFs [37–39]. Although previous research has not produced sufficient evidence for the recommendation of vitamin D among community dwelling older adults, as the number receiving in home care continues to increase in place of admission to residential aged care this may change [40, 41].

Whilst this study demonstrated good engagement of facility staff members, as fundamental to the prescription of vitamin D supplements, low GP engagement may have inhibited implementation success, as identified in this study and by others [42–44]. Poor engagement was evidenced by low survey response rates (up to 12%) and almost half (48%) of responders reporting that they did not access any study related information. This may have been the result of using fax and email to communicate and provide education, as whether GPs or pharmacists looked at the information cannot be verified. It is therefore not surprising that GP and pharmacist reported practices did not change, and only small improvements were seen in knowledge, confidence, attitudes and beliefs (Tables 5 and 8).

The majority (81%) of participating facilities initially identified that it would be feasible to engage GPs by either following up regarding individual residents (44%), raising general awareness (22%) or both (15%) (Table 6). Although around two-thirds of these sites were successful at implementing these strategies, a few frequently reported barriers were felt to have influenced the likelihood of facilities having any impact on GP practices and decisions. For example, the majority (63%) of key contacts recognised GP beliefs and attitudes regarding the evidence and their practices related to vitamin D supplementation as a barrier to implementation (Table 7). Some (17%) also felt their knowledge on the topic was a barrier, and more (29%) reflected on the difficulties experienced in their ability to directly engage with GPs.

As a number of sites foster the continuity model for residents to keep their usual GP following admission, one issue with access related to the large number of GPs who visit infrequently [45]. There were also a number of other off hand reports of issues surrounding a reliance on locum GPs, or just generally reporting that their GPs saw themselves as providing a favour in seeing their residents, rather than committing to providing ongoing care. These issues are not new knowledge, with previous surveys indicating 69% of aged care homes experience difficulty with accessing GPs [45]. From the medical practitioner perspective, annual aged care surveys evaluating member experiences of providing medical care to older Australians conducted by the Australian Medical Association highlight the difficulties experienced amongst GPs as reasons for their reduced visits to aged care homes [28].

This issue of practitioner engagement was one that differed from successful implementation efforts in Canada, where the structure by which facilities operate allowed for medical directors, pharmacists and servicing physicians among other key staff to receive education and actively engage in the action planning process [21, 46]. Few sites were able to report engaging their pharmacist at local medication advisory committee (MAC) meetings (27%), physiotherapists (5%), and one site intended on engaging with their nurse practitioner, though was unsuccessful (Table 6). Although the benefits of this role in the aged care setting are well established, there have been delays in the creation of nurse practitioner roles in aged care compared with other health care settings [47].

Lastly there was little success at implementing strategies that required organisational support, which is essential for fostering innovation and change [1, 48]. Although most (91%) surveyed staff members indicated that they agreed that vitamin D would be flagged as important for future residents, it was often not feasible to implement strategies to ensure this (Table 6). For example, although one organisation reported including vitamin D in their 30 day post admission case conference form, this had not been uploaded by the end of the intervention. Long review periods, organisational structure changes and the removal of key clinical governance groups that typically review these requests all contributed to the 0% achievement of facilities implementing this strategy. Similarly, Alamri et al. [36] reported the cumbersome process to change policies as a barrier to implementation. Although discouraged by higher level management, many sites (39%) felt they had to implement local processes, such as remembering to screen for vitamin D on admission and to discuss this at case conferences (Table 6).

Explanation for the limited uptake of certain strategies may also be the result of competing priorities, limited time and capacity as was reported by 49% of key contacts and been recognised widely in the literature as a barrier to implementation (Table 7) [15, 42, 49, 50]. One point of difference worth noting between this study and the recent Canadian trial was the access they had to multidisciplinary teams for implementing change [21, 46]. Given the existing burden on the nursing role in the Australian aged care setting, having structures where multidisciplinary teams including physicians and pharmacists can be engaged in the QI process may further facilitate implementation success [47]. Although MACs have been a proposed solution and some facilities had occasional MAC meetings, the reported attendance of GPs was low [50].

This study as any other is not without methodological limitation. It is important to be reminded that the results reported regarding compliance with QI plan implementation and identified perceived barriers are self-reported from the key contacts at each participating site. This may have underlying bias with over or underreporting, or lack in accuracy as in some cases key contacts were not directly responsible for implementation and could not confidently confirm whether certain actions had been followed up or not. Lastly, the very low survey response rates for the 12 month survey make it difficult to judge the degree to which facility staff retained the knowledge gained at the education sessions. The 12 month survey results may be influenced by non-response bias and may not be directly comparable to results from the post education session survey.

## Conclusions

This study has demonstrated success in appropriately engaging, raising awareness and improving knowledge among residential aged care facility staff, residents and their families on the importance of vitamin D supplement use for the prevention of falls. Beyond this however, important factors relating to the broader system of the residential aged care setting have been uncovered as critical to facilitating this knowledge into action.

Given the changing care needs of residents there is a need for more up to date evidence and guidelines for both the RACF and community dwelling older adult population regarding the use of vitamin D for the prevention of falls. There is also a need for more systemic support to bridge the identified GP access gap and to address the barriers concerning stakeholder beliefs about vitamin D. Without the appropriate organisational leadership, policies and procedures, vitamin D supplement use is likely to remain variable across the RACF setting.

## Data Availability

The datasets generated and analysed during the current study are available from the corresponding author on reasonable request.

## Abbreviations

ANZCTR ID: Australian New Zealand Clinical Trials Registry Trial Identification
EBP: Evidence based practice
GP: General Practitioner
IU: International Units
MAC: Medication Advisory Committee
QI: Quality Improvement
RACF: Residential aged care facility
ViDAus: Vitamin D Implementation Study
